# Gastric Th17 Cells Specific for H^+^/K^+^-ATPase and Serum IL-17 Signature in Gastric Autoimmunity

**DOI:** 10.3389/fimmu.2022.952674

**Published:** 2022-07-11

**Authors:** Chiara Della Bella, Antonio Antico, Maria Piera Panozzo, Nagaja Capitani, Luisa Petrone, Marisa Benagiano, Sofia D’Elios, Clotilde Sparano, Annalisa Azzurri, Sara Pratesi, Fabio Cianchi, Diana Ortiz-Princz, Mathijs Bergman, Nicola Bizzaro, Mario Milco D’Elios

**Affiliations:** ^1^ Department of Experimental and Clinical Medicine, University of Florence, Florence, Italy; ^2^ Laboratory of Clinical Pathology, ULSS7 Pedemontana, Hospital Alto Vicentino, Santorso, Italy; ^3^ Department of Life Sciences, University of Siena, Siena, Italy; ^4^ Endocrinology Unit, Careggi Hospital, Florence, Italy; ^5^ Department of Clinical and Experimental Medicine, University of Pisa, Pisa, Italy; ^6^ Laboratory of Clinical Pathology, Toscana Centro Hospital, Florence, Italy; ^7^ Laboratory of Molecular Microbiology, Autonomous Service Institute of Biomedicine “Dr. Jacinto Convit”, Caracas, Venezuela; ^8^ Molecular Microbiology, Faculty of Science, Vrije Universiteit Amsterdam, Amsterdam, Netherlands; ^9^ Laboratory of Clinical Pathology, San Antonio Hospital, Tolmezzo, Italy; ^10^ Laboratory of Clinical Pathology, Azienda Sanitaria Universitaria Integrata, Udine, Italy

**Keywords:** T cells, Th17, gastric autoantigen, gastric autoimmunity, gastric cancer, H^+^/K^+^-ATPase, serum IL-17, gastric mucosal immunity

## Abstract

Human gastric autoimmunity [autoimmune gastritis (AIG)] is characterized by inflammation of the gastric mucosa and parietal cell loss. The gastric parietal cell proton pump H^+^/K^+^-adenosine triphosphatase (H^+^/K^+^-ATPase) is the major autoantigen in AIG. Our work aimed to investigate the gastric H^+^/K^+^-ATPase-specific T helper 17 (Th17) responses in AIG and serum interleukin (IL)-17 cytokine subfamily in AIG patients, in healthy subjects [healthy controls (HCs)], and in patients with iron deficiency anemia (IDA) without AIG. We analyzed the activation of gastric lamina propria mononuclear cells (LPMCs) by H^+^/K^+^-ATPase and the IL-17A and IL-17F cytokine production in eight patients with AIG and four HCs. Furthermore, we compared serum levels of IL-17A, IL-17F, IL-21, IL-17E, IL-22, and IL-23 in 43 AIG patients, in 47 HCs, and in 20 IDA patients without AIG. Gastric LPMCs from all AIG patients, but not those from HCs, were activated by H^+^/K^+^-ATPase and were able to proliferate and produce high levels of IL-17A and IL-17F. AIG patients have significantly higher serum IL-17A, IL-17F, IL-21, and IL-17E (393.3 ± 410.02 pg/ml, 394.0 ± 378.03 pg/ml, 300.46 ± 303.45 pg/ml, 34.92 ± 32.56 pg/ml, respectively) than those in HCs (222.99 ± 361.24 pg/ml, 217.49 ± 312.1 pg/ml, 147.43 ± 259.17 pg/ml, 8.69 ± 8.98 pg/ml, respectively) and those in IDA patients without AIG (58.06 ± 107.49 pg/ml, 74.26 ± 178.50 pg/ml, 96.86 ± 177.46 pg/ml, 10.64 ± 17.70 pg/ml, respectively). Altogether, our results indicate that IL-17A and IL-17F are produced *in vivo* in the stomach of AIG patients following activation with H^+^/K^+^-ATPase and that serum IL-17A, IL-17F, IL-21, and IL-17E levels are significantly elevated in AIG patients but not in patients without AIG. These data suggest a Th17 signature in AIG and that IL-17A, IL-17F, IL-21, and IL-17E may represent a relevant tool for AIG management.

## Introduction

Autoimmune gastritis (AIG) is characterized by gastric corpus inflammation that does not usually result in overt disease until mucosal atrophy development and cobalamin malabsorption (1). The detection of serum anti–parietal cell autoantibodies (PCAs) without any symptoms suggests subclinical AIG (2–4). AIG is a preneoplastic condition, predisposing to the development of both gastric adenocarcinoma and type 1 neuroendocrine tumor (1, 5). The proton pump of parietal cell H^+^/K^+^-adenosine triphosphatase (ATPase) is the key autoantigen recognized in both AIG and experimental autoimmune gastritis (EAIG) (6–10). EAIG can be induced in non-thymectomized animals by immunization with either purified gastric H^+^/K^+^-ATPase or gastric mucosal extracts or by neonatal thymectomy (7–10). Inflammation of the gastric mucosa, acid-secreting parietal cell and zymogenic cell loss, and circulating anti-gastric H^+^/K^+^-ATPase autoantibodies are hallmarks of EAIG. In both AIG and EAIG, CD4^+^, CD8^+^ T cells, macrophages, and B cells are the key cells present in the gastric infiltrate (1, 2, 11).

T helper 1 (Th1) cells, secreting interferon (IFN)-γ, play a central role in AIG (12). However, emerging evidence indicates that the preferential development of Th17 mucosal responses occur in AIG also. Th17 cells, secreting IL-17 and IL-21, drive Th17 inflammation and have been implicated in the induction of many gastric inflammatory responses, including gastric cancer (13, 14). However, there are no clues on the pathogenic process mediated by H^+^/K^+^-ATPase-specific Th17 lymphocytes in human AIG.

To this aim, we investigated the *in vivo* production of IL-17A, IL-17F by gastric lamina propria mononuclear cells (LPMCs) obtained from AIG patients. As we wondered whether IL-17A, IL-17F, IL-21, IL-17E, IL-22, and IL-23 levels could be abnormal in patients with AIG, we further measured these cytokines in the serum of AIG patients, iron deficiency anemia (IDA) patients without AIG, and healthy controls (HCs).

## Materials and Methods

### Patients

Upon approval of the local ethical committee (Ethics statement n. 14936/CAM_BIO), we investigated the Th17 immune responses both at gastric and at serum level in patients with AIG (diagnosed by histology). AIG diagnosis was made according to the updated Sydney–Houston criteria, evaluating at least five random gastric biopsies with hematoxylin and eosin and Giemsa stains: two in the antrum, two in the body, and one in the angulus (15). In eight patients with AIG (six women and two men; mean age 52 years, range 37–64 years) and four healthy subjects (HCs, two women and two men; mean age 46 years, range: 42–48 years), following informed consent, biopsy specimens were obtained from the gastric mucosa in order to study LPMCs. These 8 AIG patients had both anti-intrinisic factor (IFA) and anti–parietal cell autoantibodies.

In these eight AIG patients and in another 102 patients or controls, we studied serum levels of IL-17A, IL-17F, IL-21, IL-17E, IL-22, and IL-23. Of the 110 enrolled subjects, 43 suffered from AIG (diagnosed by histology) (15), 20 (disease control) suffered from IDA and had no AIG (as defined by histology), and 47 were HCs. AIG patients were 27 (63%) women and 16 (37%) men, mean age 69.5 ± 11.2 years. The IDA patient group was composed of 17 (85%) women and 3 (15%) men, mean age 69.3 ± 22.7 years. Healthy subjects were 21 (45%) women and 26 (55%) men with a mean age of 45.0 ± 14.1 years.

Eight out of 43 AIG patients suffered also from autoimmune thyroiditis. No other autoimmune comorbidities were present in AIG, IDA, and HC subjects. None of the patients suffered from peptic ulcer, gastric cancer, gastric lymphoma nor used proton pump inhibitors in the previous 6 months. Active *H. pylori* infection, as ruled out by histopathology, was not present in any patient or control.

All AIG and IDA patients and HCs were investigated by serology (Helori CTX, Eurospital, Trieste, Italy), and three AIG patients were seropositive for *H. pylori.* All AIG patients had anti-gastric parietal cell autoantibodies detectable by indirect immunofluorescence assay on rodent tissue and/or serum intrinsic factor autoantibodies (EliA, Thermo Fisher, Uppsala, Sweden) (11, 16, 17). Sixteen out of 43 AIG patients had both anti-intrinisic factor and anti–parietal cell autoantibodies, 12 AIG patients had serum anti–parietal cell autoantibodies, and 15 AIG patients had serum autoantibodies against intrinsic factor.

### Proliferative Response to H^+^/K^+^-ATPase by Gastric Lamina Propria Mononuclear cells

Gastric specimens from eight AIG patients (having no other autoimmune disease) and four HCs were used as source of LPMCs. Specifically, LPMCs were isolated by the dithiothreitol- ethylenediaminetetraacetic acid (DTT-EDTA)-collagenase sequence (18, 19). To investigate the proliferative response to gastric H^+^/K^+^-ATPase, purified as reported (12), LPMCs were labeled with carboxyfluorescein succinimidyl ester (CFSE) (CellTrace CFSE dye, Invitrogen, USA) following manufacturer’s instructions. After CFSE staining, 2 × 10^5^ LPMCs were incubated at 37°C under 5% CO_2_ with or without gastric H^+^/K^+^-ATPase (0.3 µg/ml). Flow cytometry on BD FACS Canto II was performed using the FACSDiva software (Becton Dickinson, Franklin Lakes, NJ, USA) after 5 days of cell culture to investigate LPMC proliferation in response to H^+^/K^+^-ATPase.

### IL-17 Production by Gastric Mucosa T Cells

The IL-17 production by gastric T cells of AIG patients was investigated by both ELISpot and Fluorescence activated cell sorting (FACS) analysis. T cells of each gastric LPMC were stimulated with H^+^/K^+^-ATPase (0.3 µg/ml) or Purified Protein Derivative (PPD) (10 μg/ml) for 48 h in ELISpot microplates coated with anti–IL-17A antibody (eBioscience). At the end of the culture period, the number of IL-17 Spot Forming Cells (SFCs) was counted as described (13). For FACS analysis, we induced cytokine production by gastric isolated LPMCs, 2 × 10^5^ cells by stimulation for 6 h in 37°C with 5% CO_2_ with the Leukocyte Activation Cocktail-BD GolgiPlug™ (BD Biosciences, San Jose, CA, USA) containing phorbol 12-myristate 13-acetate (PMA), ionomycin, and brefeldin A in complete medium 5% human serum, as described (20). A basal production for each tested cytokine was determined in non-stimulated cells, treated with the Golgi block alone. T helper cell surface marker was detected by anti-human-CD4 pacific blue conjugated (e-Bioscience, Thermo Fisher Scientific, Waltham, MA, USA), and, after fixation and permeabilization with BD Cytofix/cytoperm fixation/permeabilization kit (BD Biosciences, San Jose, CA, USA), cells were stained with anti-IL-17A FITC, anti-IL-17F PerCP, following manufacturer’s instructions. Flow cytometric analysis was carried out on BD FACS Canto II using the FACSDiva software (Becton Dickinson, Franklin Lakes, NJ, USA).

### Luminex Assay for IL-17A, IL-17F, IL-21, IL-17E, IL-22, and IL-23

All sera were investigated for IL-17A, IL-17F, IL-21, IL-17E, IL-22, and IL-23 by Bio-Plex Pro™ (Bio-Rad, Hercules, CA, USA). We used Bio-Plex Manager™ software for determining the cytokine concentration. Detection range of IL-17A is 1.20–19,682.00 pg/ml; for IL-17F, it is 3.04–18,668.00 pg/ml; for IL-21, it is 8.97–147,023.00 pg/ml; for IL-17E, it is 1.00–16,375.00 pg/ml; for IL-22, it is 3.88–11,917.00 pg/ml; and for IL-23, it is 7.35–120,389.00 pg/ml.

### Statistical Analyses

The sample size required for sensitivity and specificity of the Luminex test was set in relation to the number of AIG patients admitted to the Florence reference center for AIG (21).

To calculate qualitative data, we applied descriptive statistics, as well as for standard and mean deviation. To assess the normality of each independent distribution, we used the Shapiro–Wilk test, and then the comparison was made by Mann–Whitney U test.

A p < 0.05 was considered statistically significant.

Values of cytokines below the lower limit of quantification (LLOQ) were replaced with one-half the respective LLOQ (22); no patient sample had values above the upper limit of quantification (ULOQ).

IL-17A, IL-17F, IL-21, and IL-17E Luminex assay accuracy, in terms of sensitivity and specificity, was performed by receiver operating characteristic (ROC) curve analysis. To evaluate the best cutoff, the area under the curve (AUC) and the Youden’s index (= Sensitivity ± [1 - Specificity]) were measured (23).

Statistical analyses were performed using IBM^®^SPSS Statistic version 27.0.

## Results

### Gastric T Helper Cells From Autoimmune Gastritis Patients Were Activated by H^+^/K^+^-ATPase and Were Able to Proliferate and Produce IL-17A and IL-17F

**Figure 1:**
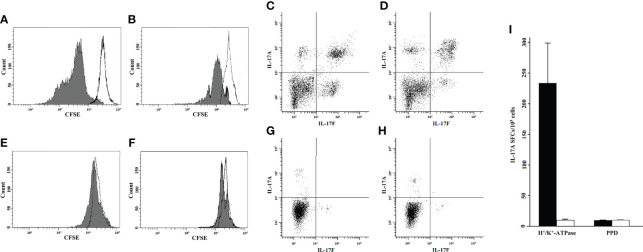
Gastric T helper cells from the lamina propria were activated by H^+^/K^+^-ATPase and were able to proliferate and produce IL-17. Proliferative response to H^+^/K^+^-ATPase, as tested by CFSE flow cytometric analysis of two representative AIG patients **(A, B)** and two healthy controls **(E, F)**. To assess CD4^+^ T-cell proliferation to H^+^/K^+^-ATPase stimulus, 5,000 events were acquired and the CFSE signal was determined on the CD4^+^ gate. Each histogram shows the merge of untreated (black line) and stimulated (gray histogram) LPMC culture condition. Intracellular cytokine analysis of IL-17A and IL-17F by gastric CD4^+^ cells of two representative AIG patients **(C, D)** and two healthy controls **(G, H)**. The 5,000 events were acquired, and IL-17A-FITC and IL-17F-PerCP signals were determined after gating for CD4^+^. Gastric mucosa T cells from each AIG patient (black histogram) or healthy control (HC) (white histogram) were stimulated with H^+^/K^+^-ATPase or PPD for 48 h in ELISpot microplates coated with anti–IL-17A antibody. At the end of the culture period, the number of IL-17A SFCs was counted **(I)**. CFSE, carboxyfluorescein diacetate succinimidyl ester; AIG, Autoimmune Gastritis; HC, Healthy Controls; SFCs, Spot Forming Cells; PPD, Purified Protein Derivative; LPMCs, Lamina propria mononuclear cells; PerCP, Peridinin-Chlorophyll-protein; FITC, Fluorescein-5-isothiocyanate; ATPase; adenosine triphosphatase.

LPMCs isolated from gastric biopsies of eight AIG patients and from four HCs were tested for their proliferative response to H^+^/K^+^-ATPase after 5 days of incubation with or without the presence of the stimulus in the medium. FACS analysis was performed, and T helper cells were identified as CD4^+^ cells and evaluated for their level of fluorescence to establish the number of generations through which a cell has progressed since the CFSE label was applied. The percentage of gastric T helper cell proliferation is summarized in **Table 1** and **Figure 1**.

**Table 1:** Percentage of T helper cell proliferation.

ID	% of CD4^+^ proliferating cells	
	Medium alone	Medium with H^+^/K^+^-ATPase
AIG-A	10.4	60.6
AIG-B	8.2	49.2
AIG-C	15.6	76.8
AIG-D	12.8	75.4
AIG-E	8.4	54.9
AIG-F	9.2	68.3
AIG-G	10.3	53.6
AIG-H	11.9	74.8
HC-I	12.7	13.4
HC-L	13.2	11.7
HC-M	9.5	10.8
HC-N	7.8	8.4

For each enrolled subject, 5,000 events were acquired and the CFSE signal was evaluated on the gated T CD4+ population.

CFSE,carboxyfluorescein diacetate succinimidyl ester.

The IL-17 production by gastric T cells of AIG patients was investigated by both ELISpot and FACS analysis. Gastric mucosa-derived T cells from each AIG patient or HC were stimulated with H^+^/K^+^-ATPase or PPD for 48 h in ELISpot microplates coated with anti–IL-17A antibody. At the end of the culture period, the number of IL-17 SFCs was counted (**Figure 1**). After specific stimulation with H^+^/K^+^-ATPase, a significant proportion of T helper cells derived from the gastric mucosa of AIG patients produced IL-17A, whereas T cells from HCs did not.

For FACS analysis, gastric T cells were stimulated for 6 h to detect IL-17A and IL-17F production. Cells were stained for membrane and intracellular markers, and the percentage of CD4^+^ T lymphocytes producing IL-17A and/or IL-17F was evaluated by FACS analysis. The results are indicated in **Table 2** and **Figure 1**.

**Table 2:** IL-17A and IL-17F production by LPMCs of 8 AIG patients and 4 HCs.

ID	% of CD4^+^ producing		
	IL-17A	IL-17F	IL-17A and IL-17F
AIG-A	8.7	4.5	13.6
AIG-B	8.2	5.4	12.8
AIG-C	11.4	5.2	15.5
AIG-D	7.3	5.6	14.8
AIG-E	9.6	7.5	16.3
AIG-F	8.0	4.6	11.4
AIG-G	7.2	5.3	14.5
AIG-H	9.3	4.8	13.6
HC-I	2.2	1.4	1.5
HC-L	1.8	0.8	0.0
HC-M	2.5	1.2	0.6
HC-N	1.4	0.5	0.0

For each sample, 5,000 events were acquired.

The percentage of CD4^+^ T cells stimulated for IL-17A and/or IL-17F production was determined by FACS analysis of intracellular cytokine antibody staining.LPMCs, Lamina propria mononuclear cells; AIG, Autoimmune Gastritis; H, Healthy Controls.

### IL-17A, IL-17F, IL-21, and IL-17E are Elevated in the Sera of Autoimmune Gastritis Patients

**Figure 2:**
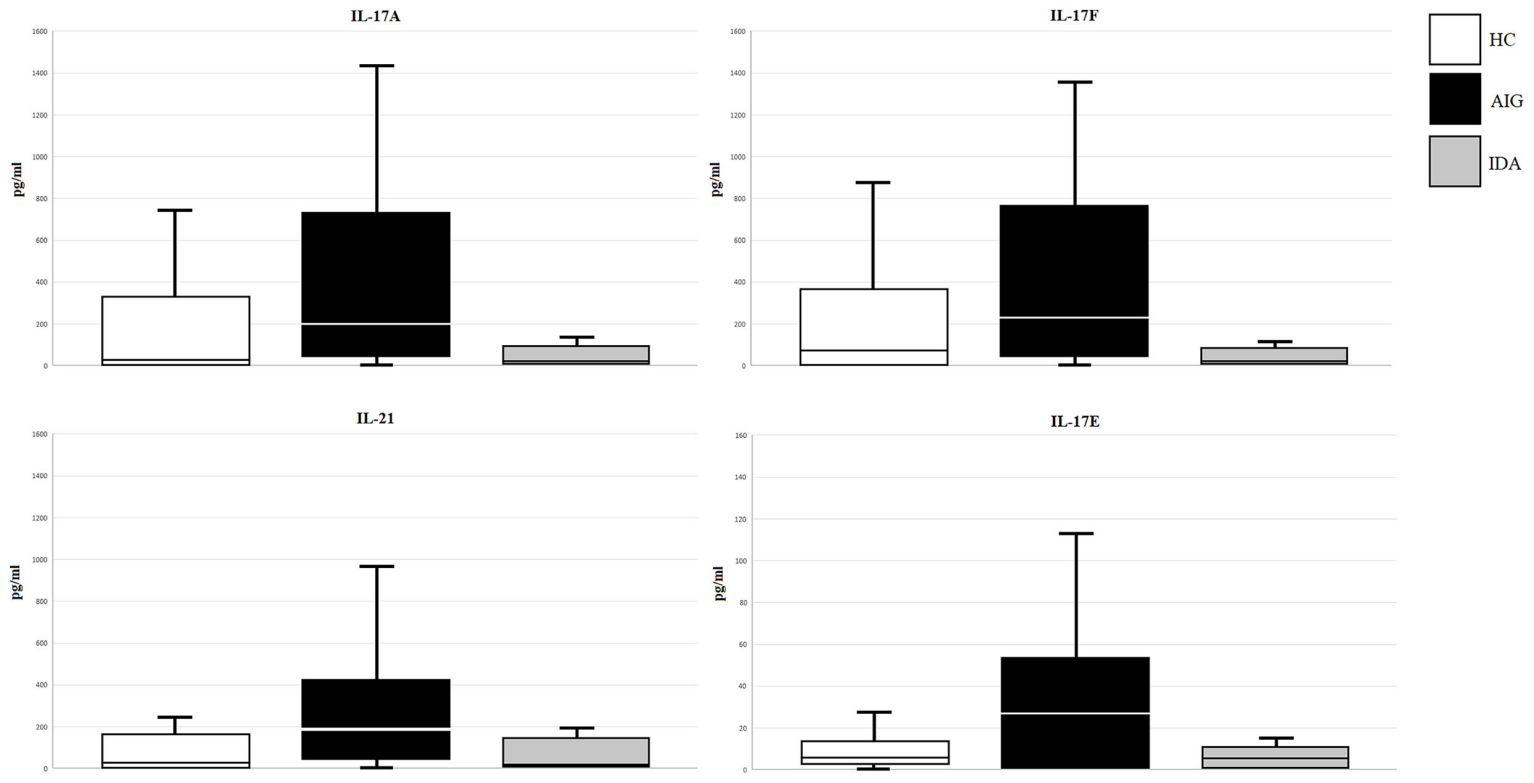
IL-17A, IL-17F, IL-21, and IL-17E (pg/ml) levels in serum samples of enrolled subjects (AIG, autoimmune gastritis; IDA, iron deficiency anemia without AIG; HC, healthy control).

Levels of serum IL-17A, IL-17F, IL-21, IL-17E, IL-22, and IL-23 were measured by Luminex assay in 43 AIG patients, 47 HCs, and 20 IDA without AIG. Multiplexed measurement of the Th17 family revealed that IL-17A, IL-17F, IL-21, and IL-17E levels increase in AIG patients compared to those in IDA patients and HCs. The total Luminex assay results are detailed in **Table 3** and **Figure 2**.

**Table 3:** Luminex assay for Th17 family cytokines.

Cytokine		Mean ± SD	p (HC vs. AIG)	p (HC vs. IDA)	p (IDA vs. AIG)
**IL-17A**	**AIG**	393.3 ± 410.02	**0.004**	0.889	**0.012**
	**HC**	222.99 ± 361.24			
	**IDA**	58.06 ± 107.49			
**IL-17F**	**AIG**	394.00 ± 378.03	**0.016**	**0.043**	**0.001**
	**HC**	217.49 ± 312.11			
	**IDA**	74.26 ± 178.50			
**IL-21**	**AIG**	300.46 ± 303.45	**0.039**	**0.035**	**<0.001**
	**HC**	147.43 ± 258.17			
	**IDA**	96.86 ± 177.46			
**IL-17E**	**AIG**	34.92 ± 32.56	**0.047**	0.276	**0.008**
	**HC**	8.69 ± 8.98			
	**IDA**	10.64 ± 17.70			
**IL-22**	**AIG**	117.60 ± 114.80	0.711	0.238	0.126
	**HC**	106.95 ± 66.88			
	**IDA**	83.66 ± 136.15			
**IL-23**	**AIG**	368.02 ± 463.52	0.645	0.810	0.728
	**HC**	275.06 ± 365.70			
	**IDA**	279.52 ± 413.86			

Significant p values are highlighted in bold.

SD, Standard deviation.

Serum samples of 43 patients with autoimmune gastritis (AIG), 47 healthy controls (HCs), and 20 patients without AIG with iron deficiency anemia (IDA) were tested for IL-17A, IL-17F, IL-21, IL-17E, IL-22, and IL-23 and between compared groups.

**Figure 3:**
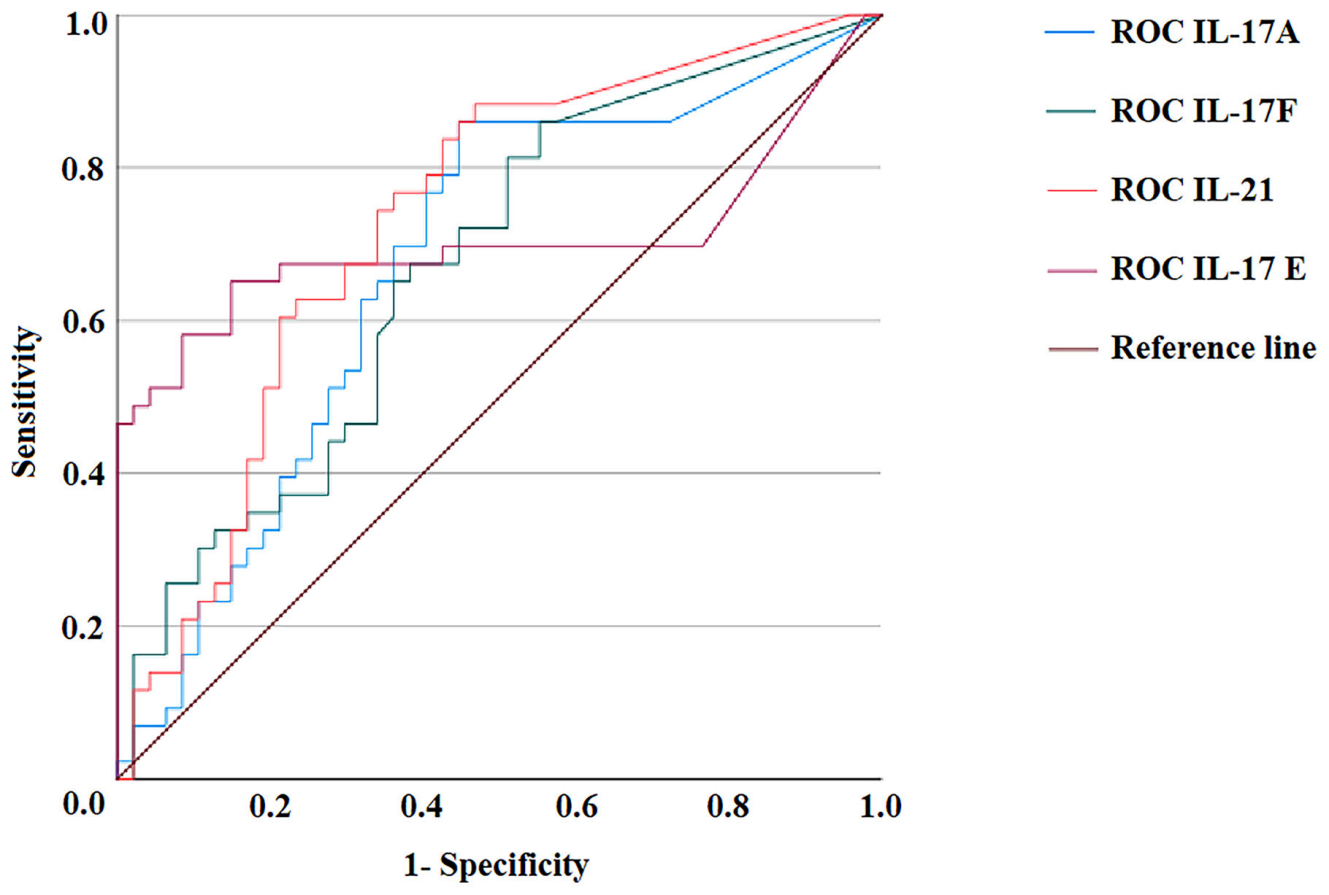
ROC curves of IL-17A, IL-17F, IL-21, and IL-17E Luminex assays. Distributions of the serum amounts of each cytokine were computed by ROC curve analysis for 47 healthy subjects and 43 autoimmune gastritis patients to assess the test’s accuracy. ROC, Receiver operating characteristic.

The ROC curve analysis was used to assess the performance of the Th17 family cytokine Luminex assay in discriminating between healthy and AIG subjects. The ROC curves for IL-17A, IL-17F, IL-21, and IL-17E are depicted in **Figure 3**. The test accuracy for each evaluated cytokine is detailed in **Table 4**.

**Table 4:** IL-17A, IL-17F, IL-21, and IL-17E Luminex assay performance.

Cytokine	AUC	Sensitivity	Specificity	Cutoff value (pg/ml)
IL-17A	0.673	86%	55%	27.1
IL-17F	0.670	86%	45%	31.45
IL-21	0.730	88%	53%	41.40
IL-17E	0.704	65%	85%	20.51

ROC curve analysis sensitivity and specificity results for each tested cytokine in 43 AIG patients and 47 HCs.

ROC, Receiver operating characteristic; AIG, Autoimmune Gastritis; HC, Healthy Controls.

## Discussion

AIG is characterized by corpus inflammation that may lead to gastric atrophy and gastric cancer. The major gastric autoantigen is H^+^/K^+^-ATPase, and gastric T-cell recognition of H^+^/K^+^-ATPase results in secretion of Th1 cytokines and activation of perforin- and FasLigand-mediated cytotoxic killing of parietal cells, leading to gastric atrophy, a preneoplastic lesion of gastric cancer (12, 24, 25). Several independent reports demonstrated the relevance of Th17 in EAIG (26–28). It was recently reported that intrinsic factor-specific T lymphocytes produce huge amounts of IL-17 in patients with AIG and pernicious anemia (PA) (11). However, it is still not clear whether the H^+^/K^+^-ATPase autoantigen might be able to drive Th17 responses in AIG.

In the current study, we found that CD4^+^ gastric LPMCs obtained from AIG patients, but not from HCs, were activated by H^+^/K^+^-ATPase and were able to proliferate and produce high levels of IL-17A and IL-17F. These findings show that not only Th1 cells but also Th17 cells specific for gastric H^+^/K^+^-ATPase drive inflammation in gastric autoimmunity (12).

To investigate the IL-17 cytokine family serum levels, we analyzed the serum levels of IL-17A, IL-17F, IL-21, IL-17E, IL-22, and IL-23 in patients with or without AIG. We found significantly higher levels of IL-17A, IL-17F, IL-21, and IL-17E in the sera of AIG patients compared to HC or IDA ones. The results obtained at both gastric and serum levels suggest that IL-17A, IL-17F, IL-21, and IL-17E are relevant cytokines for the immunopathogenesis of AIG. AIG in its late stage is characterized by intestinal metaplasia, gastric corpus and fundus dysplasia, and chromaffin cell hyperplasia that are considered precursor lesions of gastric cancer, suggesting an important link between AIG and gastric cancer (29). Ample scientific evidence indicates that gastric Th17 responses were associated with gastric adenocarcinoma and promoted gastric oncogenesis (13, 14, 30–35). In conclusion, we propose that long-lasting Th17 responses may precede the onset of gastric cancer and suggest that measurement of IL-17 family cytokines might be useful not only for the management of AIG but also for predicting the development of gastric cancer in AIG patients with gastric atrophy.

## Data Availability Statement

The raw data supporting the conclusions of this article will be made available by the authors, without undue reservation.

## Ethics Statement

The studies involving human participants were reviewed and approved by Ethical Committee Area Vasta Centro Firenze. The patients/participants provided their written informed consent to participate in this study.

## Author Contributions

CDB, and MMD’E conceived and designed the study. CDB, MPP, NC, MBen, LP, AAz, SP, SD’E, FC, CS, MBer, DO-P performed the experiments. Analysis and interpretation of data were conducted by CDB, AAn, NB, and MMD’E. CDB, AAn, NB, MBer, and MMD’E wrote, reviewed, and edited the manuscript. All the authors approved the submitted version.

## Funding

We thank the Italian Ministry of University & Research and the University of Florence for supporting our studies.

## Conflict of Interest

The authors declare that the research was conducted in the absence of any commercial or financial relationships that could be construed as a potential conflict of interest.

## Publisher’s Note

All claims expressed in this article are solely those of the authors and do not necessarily represent those of their affiliated organizations, or those of the publisher, the editors and the reviewers. Any product that may be evaluated in this article, or claim that may be made by its manufacturer, is not guaranteed or endorsed by the publisher.
